# Activation of nano-photosensitizers by Y-90 microspheres to enhance oxidative stress and cell death in hepatocellular carcinoma

**DOI:** 10.1038/s41598-022-17185-0

**Published:** 2022-07-26

**Authors:** Christopher D. Malone, Christopher Egbulefu, Alexander Zheleznyak, Jahnavi Polina, Partha Karmakar, Kvar Black, Monica Shokeen, Samuel Achilefu

**Affiliations:** 1 Department of Radiology, Mallinckrodt Institute of Radiology, Washington University School of Medicine, 4515 McKinley Ave., Floor 2, St. Louis, MO 63110 USA; 2 Department of Biomedical Engineering, Washington University in St. Louis, St. Louis, MO USA; 3 Department of Biochemistry and Molecular Biophysics, Washington University School of Medicine, St. Louis, MO USA

**Keywords:** Liver cancer, Hepatocellular carcinoma, Radiotherapy, Nanotechnology in cancer

## Abstract

While radioembolization with yttrium-90 (Y-90) microspheres is a promising treatment for hepatocellular carcinoma (HCC), lower responses in advanced and high-grade tumors present an urgent need to augment its tumoricidal efficacy. The purpose of this study was to determine whether clinically used Y-90 microspheres activate light-responsive nano-photosensitizers to enhance hepatocellular carcinoma (HCC) cell oxidative stress and cytotoxicity over Y-90 alone in vitro. Singlet oxygen and hydroxyl radical production was enhanced when Y-90 microspheres were in the presence of several nano-photosensitizers compared to either alone in cell-free conditions. Both the SNU-387 and HepG2 human HCC cells demonstrated significantly lower viability when treated with low activity Y-90 microspheres (0.1–0.2 MBq/0.2 mL) and a nano-photosensitizer consisting of both titanium dioxide (TiO_2_) and titanocene (TC) labelled with transferrin (TiO_2_-Tf-TC) compared to Y-90 microspheres alone or untreated cells. Cellular oxidative stress and cell death demonstrated a linear dependence on Y-90 at higher activities (up to 0.75 MBq/0.2 mL), but was significantly more accentuated in the presence of increasing TiO_2_-Tf-TC concentrations in the poorly differentiated SNU-387 HCC cell line (p < 0.0001 and p = 0.0002 respectively) but not the well-differentiated HepG2 cell line. Addition of TiO_2_-Tf-TC to normal human hepatocyte THLE-2 cells did not increase cellular oxidative stress or cell death in the presence of Y-90. The enhanced tumoricidal activity of nano-photosensitizers with Y-90 microspheres is a potentially promising adjunctive treatment strategy for certain patient subsets. Applications in clinically relevant in vivo HCC models are underway.

## Introduction

Hepatocellular carcinoma (HCC) is a primary liver malignancy that is the fourth leading cause of cancer-related death worldwide, and expected to further increase in Western populations with the significant rise in chronic liver disease secondary to non-alcoholic steatohepatitis^1–4^. Despite recent advances in systemic agents such as immunotherapy, there persists a substantial cohort of non-responders or poor responders to these agents, necessitating the need for therapeutic innovations^5^. Yttrium-90 (Y-90) radioembolization involves the minimally invasive, image-guided delivery of microspheres embedded with the high-energy pure beta-emitting radionuclide Y-90 to liver tumors in a precise and selective fashion directly through their arterial supply resulting in highly concentrated internal radiation therapy^6^. Effective and long-lasting objective responses are achieved when Y-90 microspheres are delivered to HCC tumors at high absorbed radiation doses^7–9^. Despite this, there are several limitations to Y-90 radioembolization, including lower response rates in patients with advanced stages of HCC, those with poorly differentiated tumors, and when adequate Y-90 radiation tumor dose is unable to be achieved either due to poor vascular conduits or significant dose heterogeneities on a micro-dosimetry level^10–14^. This highlights the need to innovate this treatment paradigm to enhance the cytotoxic efficacy of Y-90 at lower radiation doses to extend its treatment benefits to these more vulnerable and high-risk patients.

**Figure 1:**
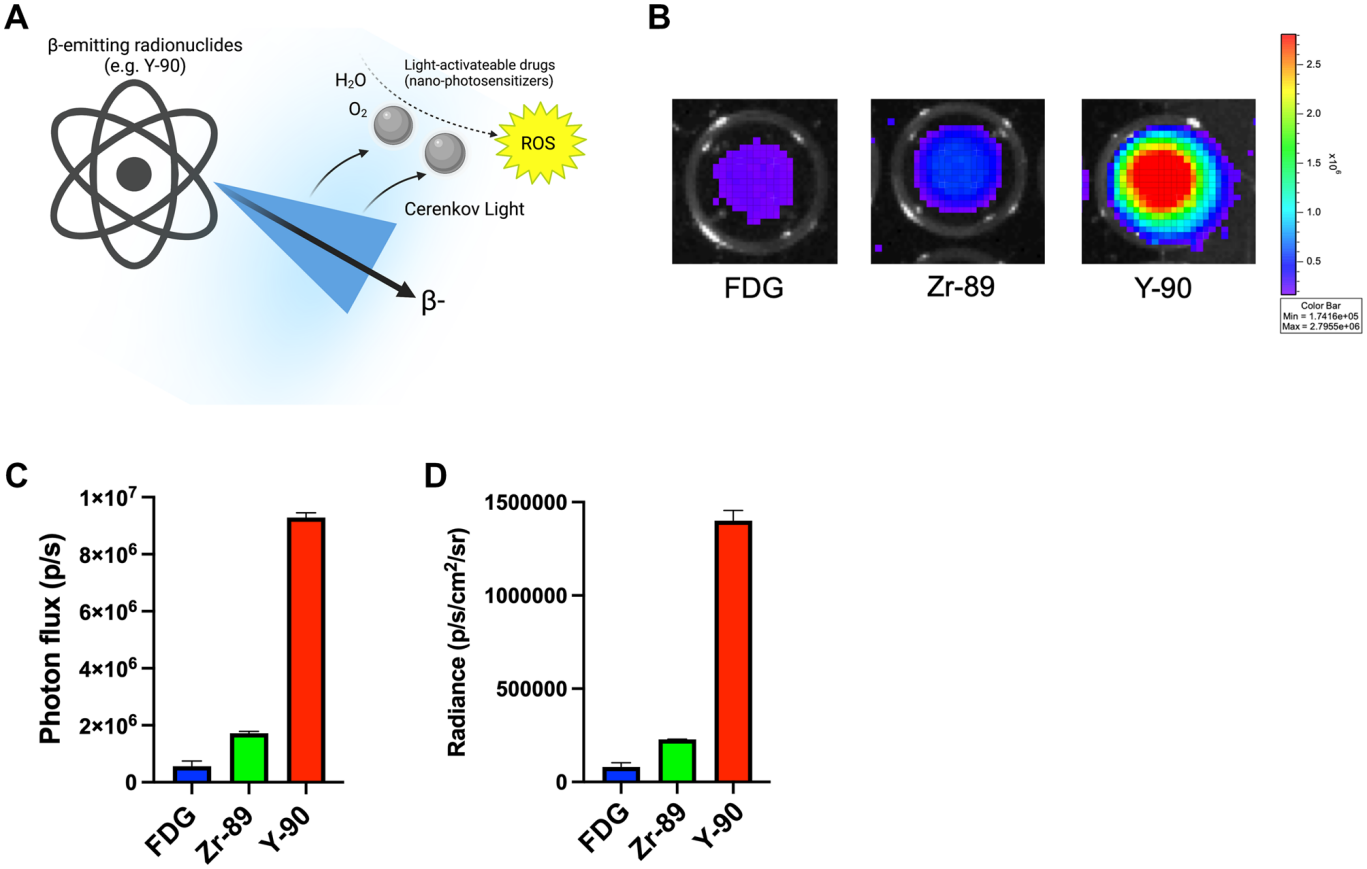
Y-90 emits an optical signal several fold higher than other beta-emitting radionuclides. (**A**) β-Emitting radionuclides such as Y-90 emit Cerenkov light that peaks in the UV-blue spectrum when charged particles (i.e. β− or β+) exceed the speed of light in a particular medium. This light, along with scintillation by the charged particle itself, can stimulate light-activatable drugs (nano-photosensitizers) to produce reactive oxygen species (ROS) from H_2_O and O_2,_ potentially introducing additional therapeutic mediators for cancer cell death. (**B**) Broad spectrum optical output of Y-90 (in Y-90Cl_3_ form) versus that of FDG and Zr-89 at equal activities (0.925 MBq) as measured on an IVIS Illumina (4 min exposure, 4 binning, 10 cm field-of-view, f-stop 1, open filter, excitation blocked). (**C**) Y-90 exhibited photon fluxes over fivefold greater than Zr-89 and over 16-fold greater than FDG. (**D**) Y-90 exhibited average radiances over sixfold greater than Zr-89 and over 17-fold greater than FDG. Values represent mean ± standard deviation of three measurements.

Beta-emitting radionuclides emit near ultraviolet to blue visible light, known as Cerenkov Radiation (CR)^15–17^. This light, along with the direct energy of the beta particle can activate light sensitive drugs known as nano-photosensitizers to generate tumoricidal reactive oxygen species (ROS) through a photodynamic therapy (PDT) process (Fig. 1A)^17,18^. In vivo, PDT results in tumor death through a multi-dimensional processes involving direct cell damage, microvascular shutdown, and activation of anti-tumor immune response through immunogenic cell death^19^. Prior work has shown that positron emitters such as F-18, Zr-89, and Ga-68 can serve as effective nano-photosensitizer activation sources to produce this “depth-independent” PDT^18,20–23^. Y-90 has been shown to be one of the brightest and most efficient CR emitters of all medically used radionuclides, which along with its high-energy beta particle, places it in an optimal position to activate nano-photosensitizers to achieve enhanced tumoricidal efficacy through a combination of both radiotherapy and PDT^24–28^.

In this study, we determined whether Y-90 in its clinically used microsphere form could activate UV-responsive nano-photosensitizers to generate tumoricidal ROS and induce HCC cell death in vitro at activities approximating absorbed radiation doses lower than typically required for HCC tumor response in the clinic according to the Medical Internal Radiation Dose (MIRD) model (< 180 Gy). Our results showed that Y-90 has a luminescence output several folds greater than that of other clinically used beta-emitters. Treatment of various nano-photosensitizers with Y-90 microspheres generated tumoricidal singlet oxygen and hydroxyl radicals in cell-free media. Using a dual nano-photosensitizer consisting of titanium dioxide (TiO_2_) and titanocene (TC) labelled with transferrin (Tf) to facilitate tumor cell uptake (TiO_2_-Tf-TC), we show Y-90 microsphere treatment activated cellular and mitochondrial oxidative stress to enhance cell death in highly malignant and poorly differentiated SNU-387 HCC cells^29^. Interestingly, well-differentiated HepG2 HCC cells showed greater cell death with Y-90 microspheres alone compared to SNU-387 cells, and this was not enhanced with nano-photosensitizers. Importantly, addition of nano-photosensitizers did not increase oxidative stress or cell death from Y-90 in normal human liver cells. These results indicate that Y-90, in its clinically used microsphere form can activate nano-photosensitizers resulting in enhanced HCC cell death with potentially greater impact in certain histologic subtypes. These results support further investigations into the use of nano-photosensitizers as an adjunctive treatment and the differential responses of Y-90 alone amongst various HCC subtypes.

## Materials and methods

### Measuring bioluminescence output of radionuclides

Y-90Cl_3_ was obtained from Eckert and Ziegler Radiopharma (Berlin, Germany). Both F-18 fluorodeoxyglucose (FDG) and Zr-89 were obtained and produced in compliance with good manufacturing practices (GMP) from a CS-15 biomedical cyclotron at our local institution. Phantoms containing 0.925 MBq of each radionuclide in triplicate were imaged in 96-well clear bottom, black walled plates, on an IVIS Illumina (PerkinElmer, Waltham, MA) with an open filter, 4 min exposure, 4 binning, 10 cm field-of-view, f-stop 1, excitation block. Broad spectrum optical output was measured using region-of-interest analysis using Living Image Software version 2.60.1 (PerkinElmer, Waltham, MA) to obtain photon flux (photon/s) and radiance (photon/s/cm^2^/sr) values.

### Nano-photosensitizer formulations

For cell-free ROS assays, hypericin (Millipore Sigma, Burlington, MA), TiO_2_ anatase 25 nM nanoparticles (Sigma Aldrich, St. Louis, MO), and titanocene (TC) formulated into human serum albumin were used as nano-photosensitizers. TiO_2_-based nano-photosensitizers were synthesized in a manner described previously^18^. Briefly, 25 nm diameter anatase TiO_2_ nanoparticles (Sigma Aldrich Co.) were suspended in deionized water to form a 10 mg/mL stock solution, which was then mixed vigorously with human apo-transferrin (Tf, Athens Research and Technology, Athens, GA), dissolved in phosphate buffered saline (PBS) using a 1:3 TiO_2_ to Tf mass ratio and dispersed by sonication at 3 W for 60 s with a Cole Parmer Ultrasonic Processor GE 130 PB (Cole Parmer, Vernon Hills, IL). The mixture was immediately filtered through a 0.22 µm polyethersulfone (PES) syringe filter (VWR Scientific, Batavia, IL), and 2% by volume of a 12 mM TC (Alfa Aesar, Tewksbury, MA) solution in DMSO was added, yielding TiO_2_-Tf-TC. Suspensions were characterized for size with dynamic light scattering (DLS) using a Malvern Zetasizer Nano ZS system (Supplemental Fig. 1). A similar protocol was used with a 12 µM Alexa680-tagged Tf (ThermoFisher Scientific, Waltham, MA) using the 1:3 TiO_2_ to Tf mass ratio to yield TiO_2_-Alexa-Tf.

HSA-TC was synthesized and characterized in the same way described previously^20^. Briefly, 80 mg TC was taken in 16 mL 0.5% aqueous solution of HSA and the mixture was shaken for 6 h at 560 oscillation per min in IKA KS 130 basic plate shaker at room temperature. The mixture was then immediately subjected to lyophilization in Thermo Fischer SAVANT RVT5105 refrigerated vapor trap lyophilizer to obtain the HSA-TC nanoparticle as orange red dry powder. The dry powders were reconstituted in DPBS immediately prior to use in the cell free assays or in cell studies. The size distributions and concentrations were confirmed in the same way as described previously^20^.

### Cell-free ROS assays

Resin Y-90 microspheres (SirSpheres, Sirtex, Woburn, MA) were obtained as unused leftover vial doses after routine clinical use and were transferred to our lab under institutional and radiation safety approval and protocol. Y-90 SirSpheres was the only form of microsphere used in this study. Both singlet oxygen and hydroxyl radical cell-free assays were performed in 96-well, clear bottom, black walled plates (Corning, Corning NY) with appropriate concentrations of nano-photosensitizers and activities of Y-90 microspheres in a total volume of 0.2 mL in phosphate-buffered saline (PBS, Life Technologies, Carlsbad, CA). For singlet oxygen radical measurement, singlet oxygen sensor green (SOSG) reagent (ThermoFisher Scientific, Waltham, MA) was added for a final concentration of 5 μM to each experimental condition well. Activation of SOSG reagent by singlet oxygen radical was determined by measuring 504 nm excitation/525 nm emission fluorescence according to manufacturer instructions using a Synergy HT multimode plate reader (BioTek Instruments Inc) immediately before and at several time points after addition of Y-90 microspheres up to 48 h. Experimental conditions included PBS alone, nano-photosensitizer alone (Hypericin 500 nM, TC 1 μM, or TiO_2_ 50 μg/mL), and Y-90 microspheres alone (0.26 MBq/0.2 mL or 0.52 MBq/0.2 mL) as controls and nano-photosensitizer (Hypericin 200 nM or 500 nM, TC 0.1 μM or 1 μM, or TiO_2_ 10 μg/mL or 50 μg/mL) added to Y-90 microspheres. The activities of 0.26 MBq and 0.52 MBq were chosen as they approximately correspond 65 Gy and 130 Gy respectively assuming Medical Internal Radiation Dose (MIRD) method in a 0.2 mL volume of space. Singlet oxygen radical scavenging was assessed by adding sodium azide (NaN_3_, Sigma Aldrich, St. Louis, MO) at a final concentration of 5 μM to Y-90 microsphere plus nano-photosensitizer conditions. Each experimental condition was repeated five times.

For hydroxyl radical measurement, hydroxyphenyl fluorescein (HPF) reagent (ThermoFisher Scientific, Waltham, MA) was added at a final concentration of 5 μM to each experimental condition well. Activation of HPF reagent by hydroxyl radical was determined by measuring 490 nm excitation/515 nm emission fluorescence according to manufacturer instructions using the above plate reader (BioTek Instruments Inc, Winooski, VT) immediately before and at several time points after addition of Y-90 microspheres up to 72 h. Experimental conditions were identical to that for singlet oxygen except with the absence of the sodium azide (NaN_3_) experimental condition. Each experimental condition was repeated five times.

### Cell culture

SNU-387 HCC cells were a generous gift from Dr. Terence Gade (Department of Radiology, University of Pennsylvania). HepG2 HCC cells were obtained from ATCC (Manassas, VA). STR profiling performed at our institution indicated that both cell lines matched reference standards per ATCC guidelines. Differentiation status of both cell lines was based off transcriptomic classifications by Caruso et al., with HepG2 cells characterized by well-differentiated, with hepatoblast-like features and SNU-387 as less-differentiated, invasive, with mesenchymal-like features^29^. SNU-387 and HepG2 cells were maintained in Roswell Park Memorial Institute (RPMI) (Gibco Life Technologies, Carlsbad, CA) and Eagle’s Minimum Essential (EMEM) media (Corning Life Sciences, Tewksbury, MA) respectively, both supplemented with 10% fetal bovine serum (Gibco) and 0.5% penicillin/streptomycin. The normal human liver epithelial cell line THLE-2 was obtained from ATCC (Manassas, VA) and maintained in Bronchial Epithelial Cell Growth media (Lonza, Basel, Switzerland) supplemented with 10% fetal bovine serum (Gibco) and 0.5% penicillin/streptomycin. All cell lines were maintained in a jacketed humidified CO_2_ (5%) incubator at 37 °C and passaged when confluent. Core facility testing at our institution for contaminants such as mycoplasma was negative.

### Internalization of TiO_2_-Tf constructs in SNU-387 cells

After grown to confluence, SNU-387 cells were plated at a density of 8000 cells/well on black-walled clear bottom 96 well plates and incubated at 37 °C for 24 h. Cells were then treated with TiO_2_-Alexa-Tf nano-photosensitizers. Both brightfield and fluorescence confocal microscopy were performed at 4, 24, and 72 h post treatment using an Olympus FV1000 confocal microscope using the AlexaFluor 633 excitation/emission channel. Fluorescence and bright-field image overlay with false color was performed using Fluoview FV10-ASW software from Olympus.

### Cell-based ROS and death assays

After grown to confluence, all cell lines were plated at a density of 8000 cells/well on black-walled clear bottom 96 well plates (Corning, Corning NY) and incubated at 37 °C for 24 h. The dual nano-photosensitizer TiO_2_-Tf-TC was then added at either 0 μg/mL (control), 10 μg/mL, or 50 μg/mL final concentration, then incubated for 2 h at 37 °C. Y-90 microspheres were then added at low or high anticipated activities. Given the marked heterogeneity and settling of Y-90 microspheres, the exact activity for each experimental well was obtained by measuring the open filter luminescence on a Synergy HT multimode plate reader (BioTek Instruments Inc, Winooski, VT) immediately after adding Y-90 microspheres and normalizing to that obtained from standard curves generated from Y-90Cl_3_ at identical plate reader gains (Supplemental Fig. 2). After 72 h of incubation at 37 °C, HPF (ThermoFisher Scientific, Waltham, MA) was added to each well for a final concentration of 5 μM to assess for hydroxyl radical. After 30 min of incubation at 37 °C, fluorescence was measured using 490 nm excitation/515 nm emission using the above plate reader. These same experimental steps and conditions were performed to assess for mitochondrial superoxide production, except after the 72 h incubation, media was suctioned off and MitoSOX Red (ThermoFisher Scientific, Waltham, MA) reagent was added at a final concentration of 5 μM according to manufacturer’s instructions. After 30 min incubation at 37 °C, fluorescence was measured using 510 nm excitation/580 nm emission. Cell death was assessed using propidium iodide (PI) staining (Millipore Sigma, Burlington, MA) according to manufacturer’s instructions using the same experimental conditions. For each nano-photosensitizer concentration, there were at least 5 experiments without Y-90 and 10 experiments with various Y-90 microsphere activities.

### HCC cell viability assays at low Y-90 activities

After grown to confluence, the two HCC cell lines (SNU-387 and HepG2) were plated at a density of 8000 cells/well on black-walled clear bottom 96 well plates (Corning, Corning NY) and incubated at 37 °C for 24 h. The dual nano-photosensitizer TiO_2_-Tf-TC was then added at either 0 μg/mL (control) or 50 μg/mL final concentration, then incubated for 2 h at 37 °C. Y-90 microspheres were then added at low (less than 0.2 MBq/0.2 mL) activities and incubated for 72 h. Exact Y-90 activity was obtained by luminescence readings in each condition with reference to Y90-Cl_3_ standards as described above. Cell viability was assessed using the Cell Titre-Glo Luminescence assay (Promega, Madison, WI) according to manufacturer’s instructions. Luminescence from Y-90 alone was accounted for by subtracting the background luminescence obtained just before adding the Cell-Titre-Glo reagent. Each experimental condition was performed in at least triplicate.

### Live-dead cell confocal microscopy in SNU-387 cells

After grown to confluence, SNU-387 cells were plated at a density of 10,000 cells/well on an 8-well chambered slide (Corning, Corning NY) and incubated at 37 °C for 24 h. Cells were then treated with either Y-90 microspheres (0.52 MBq/0.2 mL) alone, TiO_2_-Tf-TC (50 μg/mL) alone, Y-90 microspheres combined with TiO_2_-Tf-TC, or untreated for 72 h. Following this, a live/dead cell stain consisting of Cyto-dye to stain live cells and propidium iodide (PI) to stain dead cells (Millipore Sigma, Burlington, MA) was added to each condition and incubated at 37 °C for 10 min according to manufacturer's instructions. The media was suctioned off, washed with PBS, and the cells were fixed with para-formaldehyde (Sigma Aldrich, St. Louis, MO). Cells were then stored at 4 °C until the Y-90 had fully decayed (greater than 10 half-lives, approximately 30 days). Live and dead cell fluorescence confocal microscopy was performed using 488 nm excitation/518 nm emission and 488 nm excitation/632 nm emission respectively according to manufacturer’s instructions using an Olympus FV1000 confocal microscope. Fluorescence and bright-field image overlay with false color was performed using Fluoview FV10-ASW software from Olympus.

### Statistical analysis

Statistical analysis was performed using GraphPad Prism 9.2 (GraphPad Software, Inc., La Jolla, CA). All data for radionuclide optical output, cell-free ROS production, and cell viability is expressed as assay signal in mean ± standard deviation. Statistical significance between experimental groups was assessed using one-way ANOVA followed by Tukey’s multiple comparison tests. Cell-based HPF, MitoSox, and PI values were plotted versus Y-90 microsphere activity for each nano-photosensitizer concentration. Simple linear regression was performed for each nano-photosensitizer concentration, and differences in slopes were determined using one-way ANOVA. Statistical significance was set as alpha less than 0.05.

## Results

### Y-90 exhibited high luminescence output and reactive oxygen species (ROS) production that was markedly enhanced with nano-photosensitizers and Y-90 microspheres in cell-free conditions

Broad spectrum luminescence demonstrated that Y-90 had a bright optical output several times greater than F-18 or Zr-89. Phantoms containing equal activities (0.925 MBq) of F-18 fluorodeoxyglucose (FDG), Zr-89, and Y-90 (in Y-90Cl_3_ form) were imaged on an IVIS Illumina (4 min exposure, 4 binning, 10 cm field-of-view, f-stop 1, open filter, excitation blocked). As shown in Fig. 1, Y-90 optical luminescence was over fivefold higher compared to Zr-89 and over 16-fold high compared to F-18 (Fig. 1B), as measured by both photon flux (photon/s) (Fig. 1C) and radiance (photon/s/cm^2^/sr) (Fig. 1D), in keeping with prior calculations and measurements^30^.

**Figure 2:**
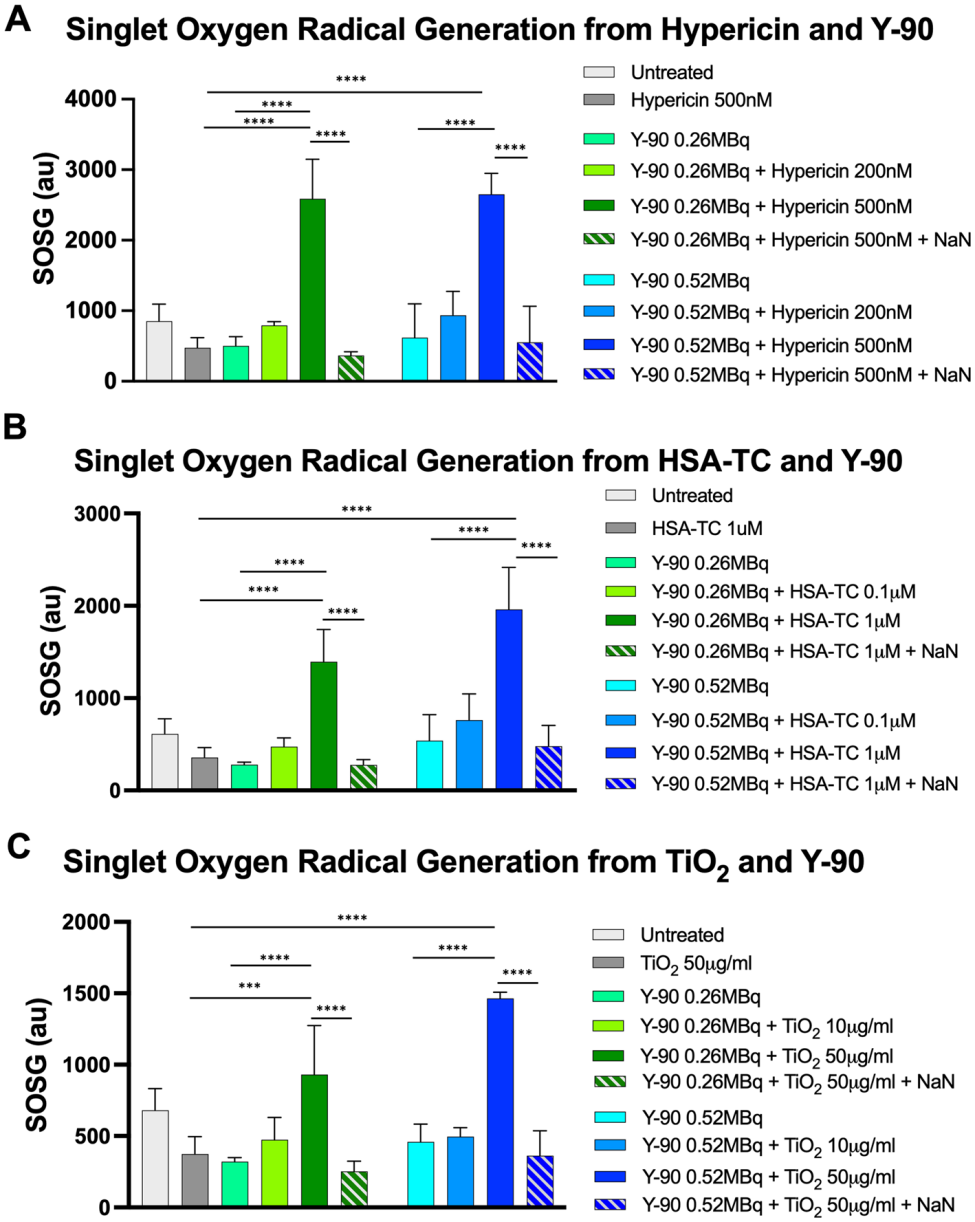
Combining Y-90 microspheres with nano-photosensitizers results in enhanced singlet oxygen radical production in cell-free media. Marked increase in singlet oxygen radial production was seen in the presence of nano-photosensitizers (**A**) hypericin 500 nM, (**B**) titanocene bound to human serum albumin (TC) 1 μM, and (**C**) TiO_2_ 50 μg/mL with both low and high Y-90 microsphere activities compared to either alone. This effect was diminished in the presence of the singlet oxygen radical scavenger sodium azide (NaN_3_). Values represent mean ± standard deviation of five measurements. ***p < 0.001, ****p < 0.0001.

**Figure 3:**
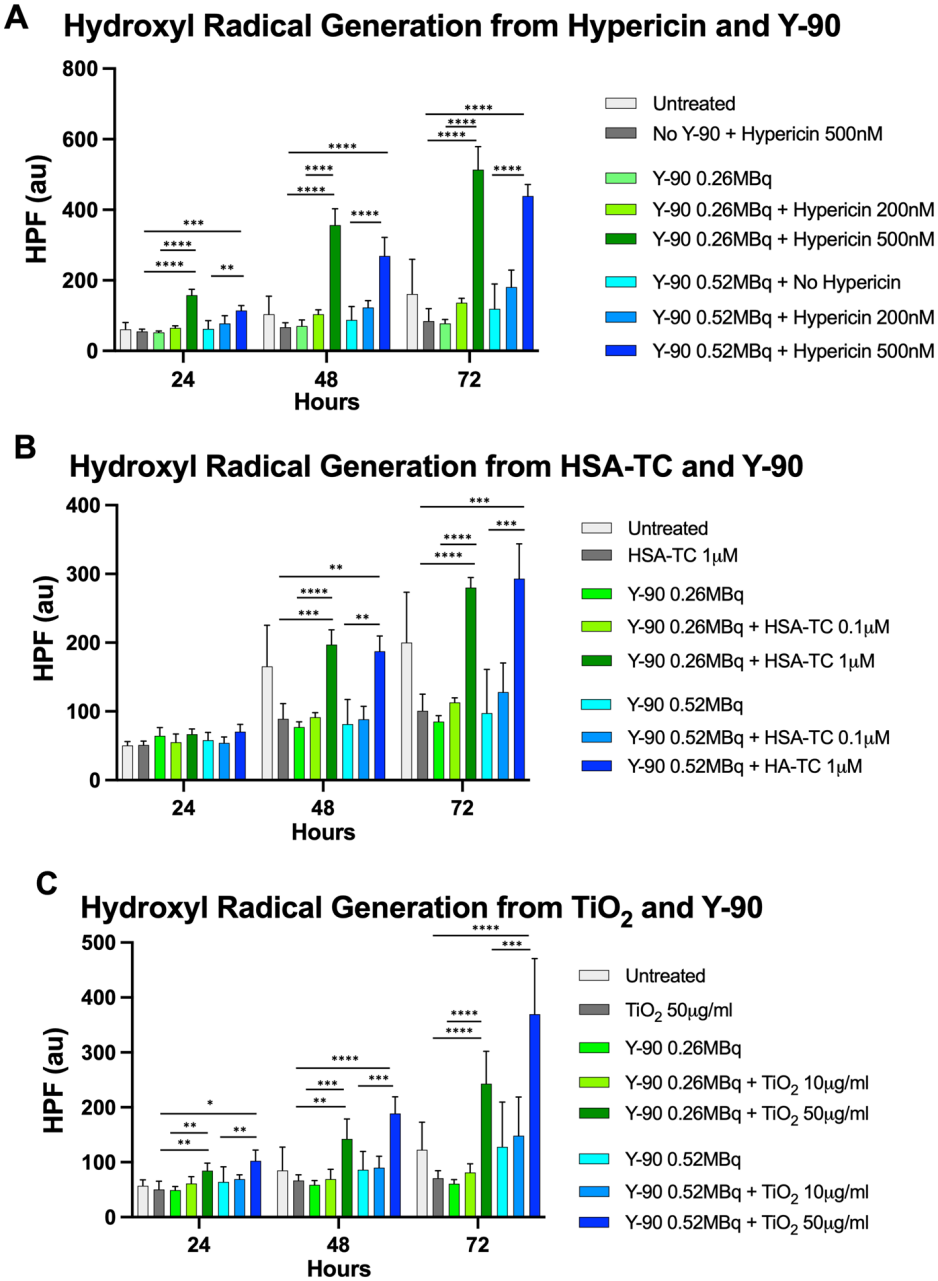
Combining Y-90 microspheres with nano-photosensitizers results in enhanced hydroxyl radical production over time in cell-free media. Marked increase in hydroxyl radical production was see in the presence of nano-photosensitizers (**A**) hypericin 500 nM, (**B**) titanocene bound to human serum albumin (TC) 1 μM, and (**C**) TiO_2_ 50 μg/mL with both low and high Y-90 microspheres activities compared to either alone. This effect became more pronounced over time. Values represent mean ± standard deviation of five measurements. *p < 0.05, **p < 0.01, ***p < 0.001, ****p < 0.0001.

Generation of singlet oxygen radical, the primary tumoricidal mediator of photodynamic therapy (PDT), was assessed using the singlet oxygen sensor green (SOSG) assay with and without different Y-90 microsphere activities (0.26–0.52 MBq/0.2 mL, approximating 65–130 Gy assuming MIRD) and nano-photosensitizer concentrations (Fig. 2). Significant increase in singlet oxygen radical production was seen with higher nano-photosensitizer concentrations (over threefold with Hypericin 500 nM Fig. 2A, over twofold with TC 1 μM, Fig. 2B; and up to twofold with TiO_2_ 50 μg/mL, Fig. 2C) when combined with both low (0.26 MBq/0.2 mL) and higher (0.52 MBq/0.2 mL) activities of Y-90 microspheres after 48-h treatment (all p < 0.0001). This effect was abolished in the presence of the singlet oxygen radical scavenger sodium azide (NaN_3_), indicating that this signal was indeed due to ROS production^31^. Generation of hydroxyl radical over time was assessed using the hydroxyphenyl fluorescein (HPF) assay with and without various activities of Y-90 microspheres and concentrations of nano-photosensitizers. As shown in Fig. 3, increased hydroxyl radical was seen with higher concentrations of each nano-photosensitizer and Y-90 microsphere activities and increased over time up to 72 h (just over one Y-90 half-life). Similar to singlet oxygen production, hydroxyl radical production from hypericin or TC did not appear to be influenced by higher Y-90 microsphere activities (Fig. 3A,B). However, TiO_2_ generation of hydroxyl radical was increased at higher Y-90 microsphere activities (Fig. 3C). Neither singlet oxygen or hydroxyl radical was significantly generated by Y-90 microspheres or nano-photosensitizer alone in cell-free media (Figs. 2 and 3), similar to other results utilizing therapeutic beta-emitting radionuclides such as I-131 as a platform for PDT^32^. Overall, this data demonstrated that Y-90 exhibited high luminescence output and reactive oxygen species (ROS) production was markedly enhanced with nano-photosensitizers and Y-90 microspheres in cell-free conditions.

### Cellular oxidative stress from Y-90 microspheres is enhanced in the presence of nano-photosensitizers in poorly-differentiated SNU-387 but not well-differentiated HepG2 HCC cells

**Figure 4:**
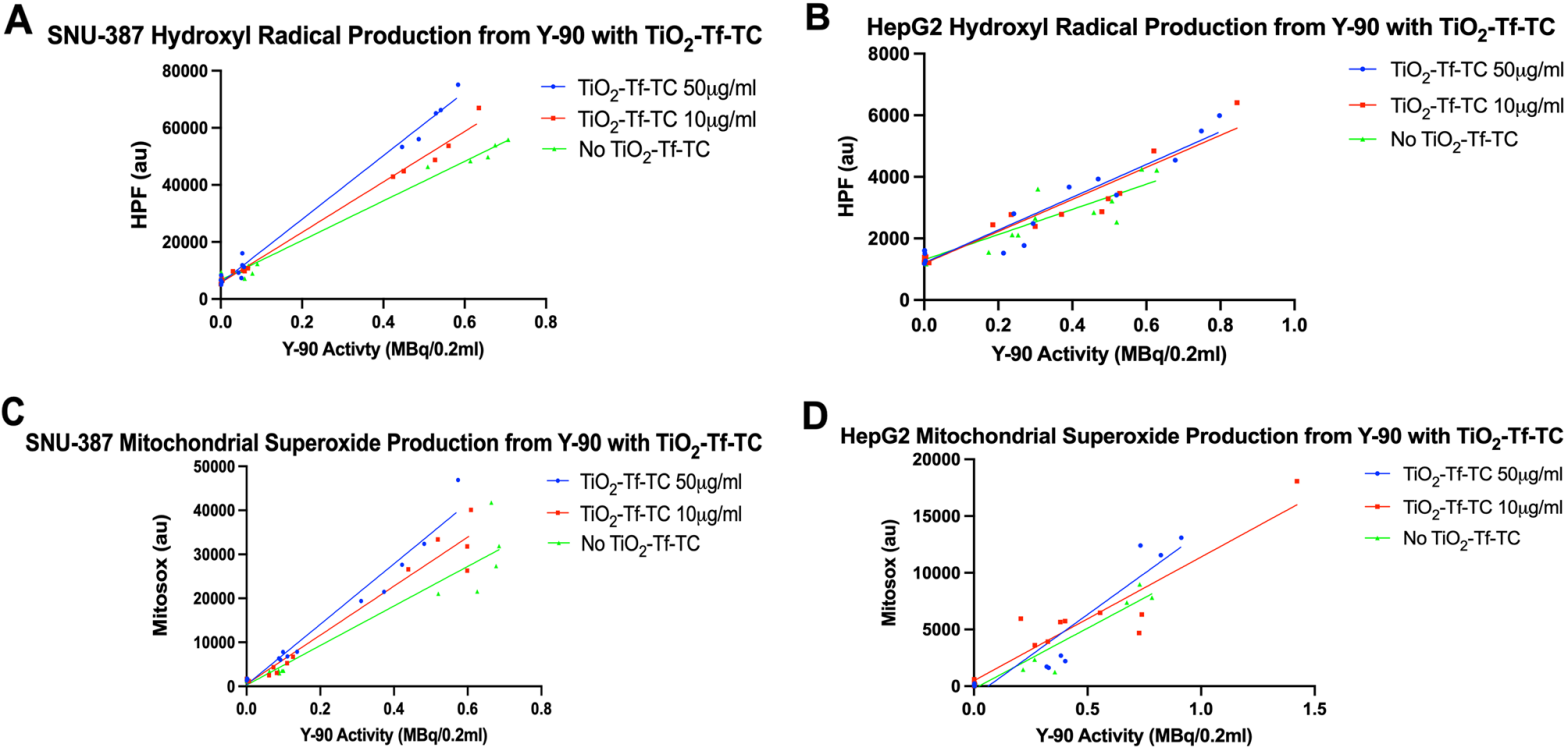
HCC cellular oxidative stress from Y-90 microspheres is increased when combined with nano-photosensitizers at 72 h in SNU-387 but not HepG2 cells. (**A**) The production of hydroxyl radical in the SNU-387 HCC cell line is linearly dependent on Y-90 microsphere activity but is more pronounced in the presence of increasing concentrations of the dual targeted nano-photosensitizer TiO_2_-Tf-TC (p < 0.0001). (**B**) However, no difference in hydroxyl radical production was seen in well-differentiated HepG2 cells with the addition of TiO_2_-Tf-TC (p = 0.1744). (**C**) Similarly, the production of mitochondrial superoxide in SNU-387 cells is also linearly dependent on Y-90 microsphere activity and is more pronounced in the presence of increasing concentrations of TiO2-Tf-TC (p = 0.0002). (**D**) However, no difference in mitochondrial superoxide production was seen in HepG2 cells with the addition of TiO_2_-Tf-TC (p = 0.1087).

For experiments involving HCC cells in vitro, we utilized a transferrin (Tf) labelled dual nano-photosensitizer consisting of both TiO_2_ and TC (TiO_2_-Tf-TC), given previous work demonstrating greater tumoricidal efficacy compared to single nano-photosensitizers with lower radiance radionuclides^18^. In addition, transferring receptor (TfR) has found to be overexpressed in HCC and prior work with Tf-labelled nanoparticles showed high accumulation within HCC tumors versus non-targeted formulations in vivo^33,34^. We showed that this nano-photosensitizer construct labelled with AlexaFluor-680 internalized within SNU-387 HCC cells (Supplemental Fig. 3). Cellular oxidative stress as assessed by hydroxyl radical and mitochondrial superoxide production in both poorly-differentiated SNU-387 and well-differentiated HepG2 HCC cells at 72 h was tested using the HPF and Mitosox assays respectively with various Y-90 microsphere activities and increasing concentrations of TiO_2_-Tf-TC. Analysis at 72-h was chosen given the significant increase in hydroxyl radical generation in cell-free media (Fig. 3) at this time point and it was just over one half-life of Y-90. As shown in Fig. 4A,B, hydroxyl radical generation was increased in the presence of Y-90 microspheres alone without TiO_2_-Tf-TC and demonstrated a linear dependence on Y-90 activity (Fig. 4A, increase of 69,337 HPF a.u. per Y-90 MBq/0.2 mL, 95% CI 65,145–73,529, r^2^ = 0.99 for SNU-387 cells and Fig. 4B, increase of 4094 HPF a.u per Y-90 MBq/0.2 mL, 95% CI 2932–5255, r^2^ = 0.92 for HepG2 cells). In poorly differentiated SNU-387 cells, conditions with increasing concentrations of TiO_2_-Tf-TC showed significantly greater hydroxyl radical generation in the presence of Y-90 microspheres compared to that without TiO_2_-Tf-TC, and was linearly related with Y-90 activity (Fig. 4A, increase of 88,426 HPF a.u. per Y-90 MBq/0.2 mL, 95% CI 83,815–93,037 for TiO_2_-Tf-TC 10 μg/mL, and increase of 111,557 HPF a.u. per Y-90 MBq/0.2 mL, 95% CI 105,464–117,650 for TiO_2_-Tf-TC 50 μg/mL, both r^2^ = 0.99, p < 0.0001), indicating that TiO_2_-Tf-TC enhanced oxidative stress in HCC cells in vitro. Interestingly, the addition of TiO_2_-Tf-TC to the well differentiated HepG2 cells did not result in a significant increase in hydroxyl radical generation (Fig. 4B, increase of 5209 HPF a.u. per Y-90 MBq/0.2 mL, 95% CI 83,815–93,037, r^2^ = 0.92 for TiO_2_-Tf-TC 10 μg/mL, and increase of 5327 HPF a.u. per Y-90 MBq/0.2 mL, 95% CI 105,464–117,650, r^2^ = 0.91 for TiO_2_-Tf-TC 50 μg/mL, p = 0.1744).

Production of mitochondrial superoxide in SNU-387 cells was also increased in the presence of Y-90 microspheres alone without TiO_2_-Tf-TC in a linear relationship with Y-90 activity (Fig. 4C, increase of 44,941 MitoSox a.u. per Y-90 MBq/0.2 mL, 95% CI 37,002–52,880, r^2^ = 0.92). Similar to the observation with hydroxyl radical generation, mitochondrial superoxide generation in SNU-387 cells was significantly enhanced with increasing concentrations of TiO_2_-Tf-TC (Fig. 4C, increase of 55,698 MitoSox a.u. per Y-90 MBq/0.2 mL, 95% CI 48,665–62,732, r^2^ = 0.96 for TiO_2_-Tf-TC 10 μg/mL, and increase of 68,740 MitoSox a.u. per Y-90 MBq/0.2 mL, 95% CI 60,940–76,540 r^2^ = 0.97 for TiO_2_-Tf-TC 50 μg/mL, p = 0.0002), potentially indicating mitochondrial oxidative stress a therapeutic byproduct of Y-90 alone that is further enhanced when combined with nano-photosensitizers in this cell line^35^. While mitochondrial superoxide production was also linearly related to Y-90 activity in HepG2 cells, it was not significantly enhanced when adding nano-photosensitizer (Fig. 4D, increase of 10,690 MitoSox a.u. per Y-90 MBq/0.2 mL, 95% CI 8459–12,922, r^2^ = 0.9288 for Y-90 without TiO2-Tf-TC, increase of 10,910 MitoSox a.u. per Y-90 MBq/0.2 mL, 95% CI 8319–13,501, r^2^ = 0.8752 for TiO_2_-Tf-TC 10 μg/mL, and increase of 14,452 MitoSox a.u. per Y-90 MBq/0.2 mL, 95% CI 10,895–18,009, r^2^ = 0.8913 for TiO_2_-Tf-TC 50 μg/mL, p = 0.1087). Taken together, these data showed that the cellular oxidative stress from Y-90 microspheres was enhanced in the presence of nano-photosensitizers in SNU-387 but not HepG2 HCC cells, suggesting differential effects based on histological subtypes.

### Nano-photosensitizers enhance Y-90 microsphere-mediated HCC cytotoxicity

**Figure 5:**
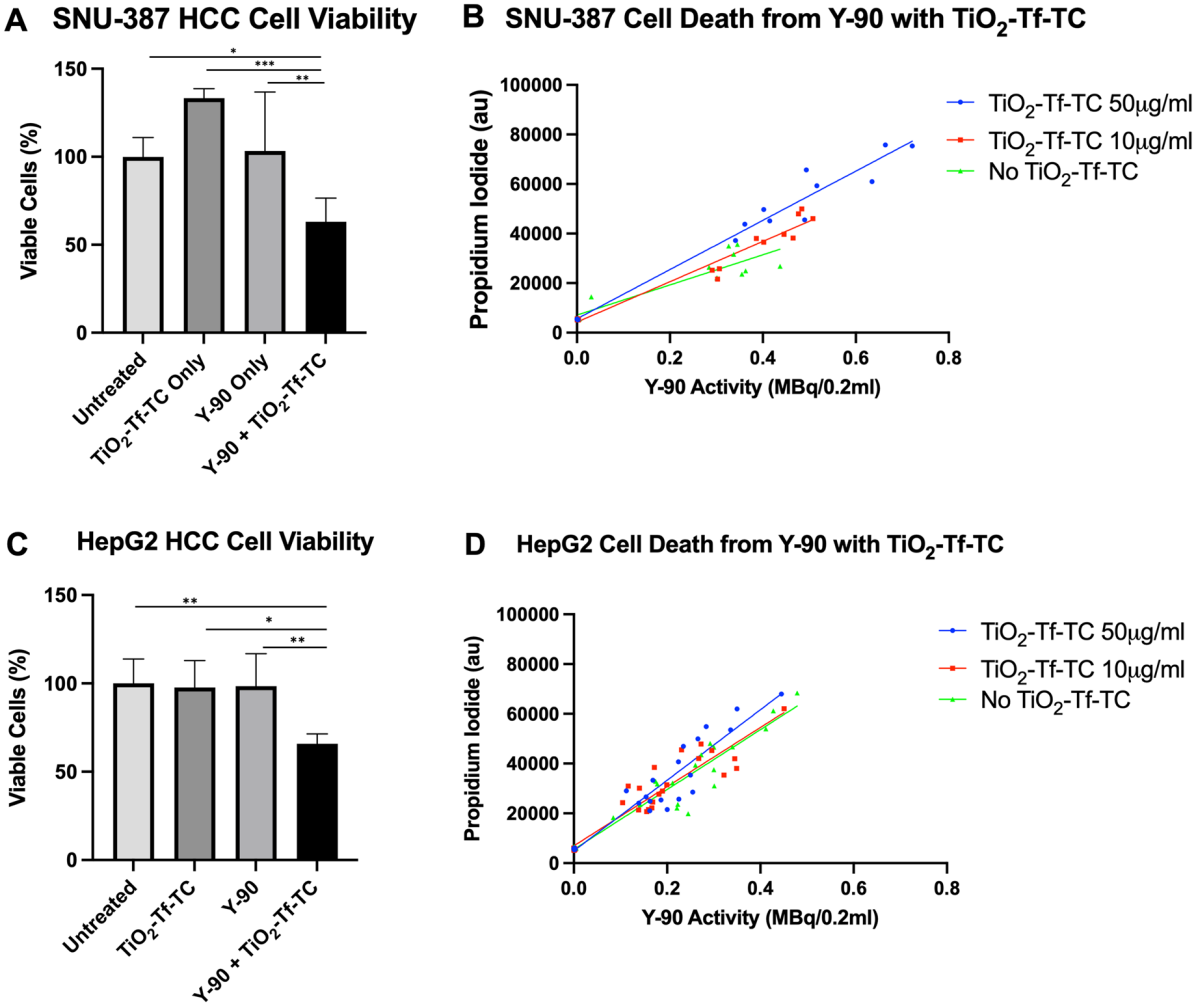
HCC cytotoxicity from Y-90 microspheres is increased when combined with nano-photosensitizers at 72 h. (**A**) SNU-387 cell viability, as measured by ATP quantitation is decreased with the combination of TiO_2_-Tf-TC and Y-90 microspheres at an activity that does not cause decrease in cell viability with Y-90 alone (0.1–0.2 MBq/0.2 mL) (p = 0.0002). (**B**) SNU-387 cell death, as measured by propidium iodide uptake, is linearly dependent on Y-90 microsphere activity but is more pronounced in the presence of increasing concentrations of TiO_2_-Tf-TC (p = 0.0002). (**C**) HepG2 cells also showed decreased viability with the combination of TiO_2_-Tf-TC and Y-90 microspheres at low activities (p = 0.0035). (**D**) However, TiO_2_-Tf-TC did not result in enhanced cell death in HepG2 cells at higher Y-90 activities as assessed by propidium iodide uptake (p = 0.0886). Of note, the degree of cell death per Y-90 activity alone was more significant with HepG2 cells than it was for the more poorly differentiated SNU-387 cells (p < 0.0001). *p < 0.05, **p < 0.01, ***p < 0.001 for ANOVA multiple comparisons in (**A**) and (**C**).

We sought to determine whether TiO_2_-Tf-TC would induce HCC cell death in combination with otherwise non-toxic Y-90 microsphere activities. To test this, we determined cell viability of SNU-387 and HepG2 cells by ATP quantification treated with TiO_2_-Tf-TC with and without low activities of Y-90 microspheres (0.1–0.2 MBq/0.2 mL). Both cell lines demonstrated a significant decrease in cell viability with the combination of Y-90 microspheres and TiO_2_-Tf-TC after 72 h treatment but not with either alone, compared to untreated cells (Fig. 5A, p = 0.0002 for SNU-387 cells, Fig. 5C, p = 0.0035 for HepG2 cells). At higher Y-90 activities (up to 0.75 MBq/0.2 mL), we determined cell death utilizing propidium iodide (PI) staining given the increased interference from Y-90’s Cerenkov emission with luminescence-based cell-viability assays. As demonstrated in Fig. 5B, SNU-387 cell death was linearly dependent on Y-90 activity (increase of 60,793 PI a.u. per Y-90 MBq/0.2 mL, 95% CI 44,126–77,459, r^2^ = 0.84), similar to the cell-based hydroxyl radical and mitochondrial superoxide findings. However, conditions with increasing concentrations of TiO_2_-Tf-TC showed significantly greater SNU-387 cell death in the presence of Y-90 microspheres compared to that without TiO_2_-Tf-TC, and was also linearly related with Y-90 activity (Fig. 5B, increase of 81,450 PI a.u per Y-90 MBq/0.2 mL, 95% CI 71,662–91,238, r^2^ = 0.96 for TiO_2_-Tf-TC 10 μg/mL and increase of 99,371 PI a.u. per Y-90 MBq/0.2 mL, 95% CI 88,702–110,039, r^2^ = 0.97 for TiO_2_-Tf-TC 50 μg/mL, p = 0.0002). Live/dead cell stain confocal microscopy of untreated SNU-387 cells or those treated with TiO_2_-Tf-TC alone (50 μg/mL) showed mostly live cells (green Cyto-dye stain, Supplemental Fig. 4). In contrast, cells treated with Y-90 microspheres alone showed minimal live cells remaining, while those treated with combined Y-90 microspheres and TiO_2_-Tf-TC showed complete absence of live cells, presence of necrotic cells (red PI stain), and an abundance of cellular debris (Supplemental Fig. 4). HepG2 cells demonstrated a linear and increased rate of cell death with Y-90 activity alone compared to SNU-387 cells (Fig. 5D, increase of 120,334 PI a.u. per Y-90 MBq/0.2 mL, 95% CI 106,360–134,309, r^2^ = 0.9234 for HepG2 vs increase of 60,793 PI a.u. per Y-90 MBq/0.2 mL, 95% CI 44,126–77,459, r^2^ = 0.8404 for SNU-387 cells, p < 0.0001). Similar to that observed with hydroxyl radical and mitochondrial superoxide production, cell death was not enhanced with the addition of nano-photosensitizers in HepG2 cells (Fig. 5D, increase of 118,912 PI a.u per Y-90 MBq/0.2 mL, 95% CI 102,727–135,097, r^2^ = 0.89 for TiO_2_-Tf-TC 10 μg/mL and increase of 141,732 PI a.u. per Y-90 MBq/0.2 mL, 95% CI 123,651–159,814, r^2^ = 0.9009 for TiO_2_-Tf-TC 50 μg/mL, p = 0.0886). Overall, we demonstrated that nano-photosensitizers significantly enhanced Y-90 microsphere-mediated HCC cytotoxicity.

### Nano-photosensitizers do not increase oxidative stress or cell death in normal liver epithelial cells

**Figure 6:**
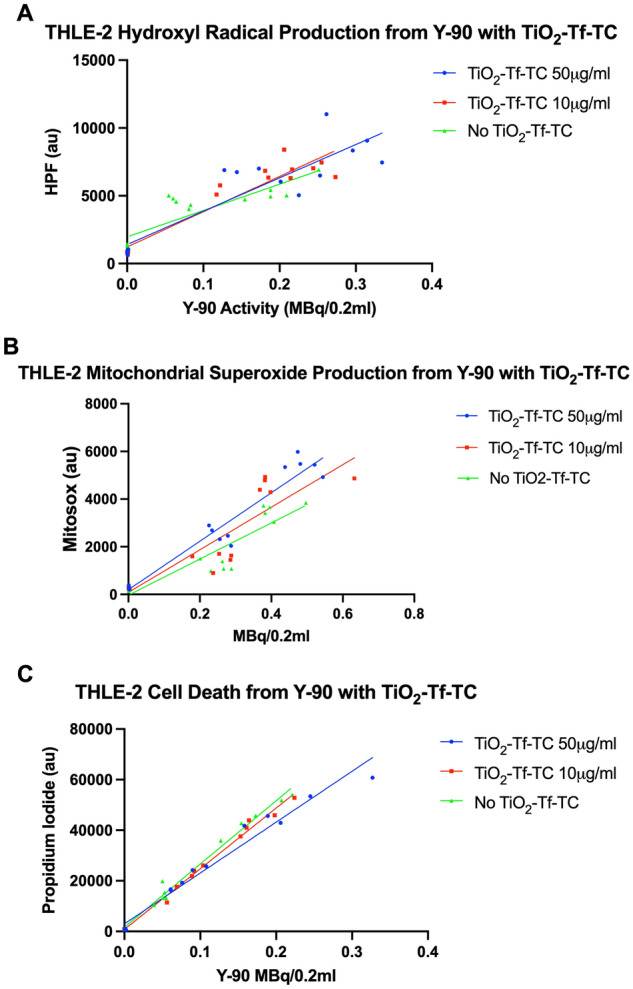
Nano-photosensitizers do not increase oxidative stress or cell death in normal human hepatocytes. The production of (**A**) hydroxyl radical, (**B**) mitochondrial superoxide, and (**C**) cell death in THLE-2 cells was linearly dependent on Y-90 activity but was not increased in the presence of TiO_2_-Tf-TC.

We sought to determine whether TiO_2_-Tf-TC increased oxidative stress and cell death in normal human liver cells, which would have implications with added unwanted toxicities from this combined treatment. As shown in Fig. 6A, there was a linear relationship with Y-90 activity and hydroxyl radical generation in THLE-2 cells, but this was not increased when co-treating with increased concentrations of TiO_2_-Tf-TC (increase of 24,593 HPF a.u. per Y-90 MBq/0.2 mL, 95% CI 17,438–31,748, r^2^ = 0.81 for Y-90 without TiO_2_-Tf-TC, increase of 26,016 HPF a.u. per Y-90 MBq/0.2 mL, 95% CI 20,808–31,224, r^2^ = 0.90 for TiO_2_-Tf-TC 10 μg/mL, and increase of 24,593 HPF a.u. per Y-90 MBq/0.2 mL, 95% CI 17,438–31,748, r^2^ = 0.81 for TiO_2_-Tf-TC 50 μg/mL, p = 0.387). While there was a trend towards increased mitochondrial superoxide production in THLE-2 cells with addition of TiO_2_-Tf-TC, this was not statically significant (Fig. 6B, increase of 7590 MitoSox a.u. per Y-90 MBq/0.2 mL, 95% CI 5640–9540, r^2^ = 0.84 for Y-90 without TiO_2_-Tf-TC, increase of 8935 MitoSox a.u. per Y-90 MBq/0.2 mL, 95% CI 6240–11,630, r^2^ = 0.80 for TiO_2_-Tf-TC 10 μg/mL, and increase of 10,175 MitoSox a.u. per Y-90 MBq/0.2 mL, 95% CI 8606–11,743, r^2^ = 0.94 for TiO_2_-Tf-TC 50 μg/mL, p = 0.1923). While there was also an expected linear relationship with THLE-2 cell death and Y-90 activity, this was not increased when adding TiO_2_-Tf-TC (Fig. 6C, increase of 250,102 PI a.u per Y-90 MBq/0.2 mL, 95% CI 233,598–266,606, r^2^ = 0.99 for Y-90 without TiO_2_-Tf-TC, increase of 239,867 PI a.u. per Y-90 MBq/0.2 mL, 95% CI 227,952–251,782, r^2^ = 0.99 for Y-90 with TiO_2_-Tf-TC 10 μg/mL, and increase of 201,086 PI a.u per Y-90 MBq/0.2 mL, 95% CI 180,258–221,914, r^2^ = 0.97 for Y-90 with TiO_2_-Tf-TC 50 μg/mL). As expected, nano-photosensitizers did not increase oxidative stress or cell death in normal liver epithelial cells.

## Discussion

Given that Y-90 is one of the brightest and most efficient Cerenkov emitters of clinically used beta-emitting radionuclides, we hypothesized that it would also be a viable activator of nano-photosensitizers to enhance tumoricidal activity of HCC^24^. Here we demonstrate that Y-90, specifically in its clinically used microsphere form (SirSpheres), activates light sensitive nano-photosensitizers to generate tumoricidal singlet oxygen radical among other ROS in cell-free media, which is a key mediator of PDT. Furthermore, this resulted in differential effects in HCC cell lines in vitro, with increased cellular hydroxyl and mitochondrial superoxide production along with enhanced cytotoxicity in the poorly differentiated SNU-387 HCC cell line but not in the well differentiated HepG2 HCC cell line. This corroborates prior work demonstrating that low radiance beta-emitters such as F-18, Zr-89, and Cu-64 can activate nano-photosensitizers to enable depth-independent PDT to treat various malignancies^18,20,21^. Furthermore, while some beta-emitters such as Ga-68 demonstrate comparable Cerenkov radiance to Y-90, the latter has the therapeutic advantage of a longer half-life (68 min vs. 64.1 h) and high energy beta-minus emission enabling concurrent radiotherapy^22^.

The addition of various nano-photosensitizers to Y-90 microspheres resulted in substantial generation of the highly tumoricidal singlet oxygen radical, indicating that this radionuclide can be a potential source of PDT with a variety of light-activatable compounds in nature. This is in keeping with modeling and simulations performed by others predicting that Y-90 may be an ideal Cerenkov light source for PDT^36,37^. It also adds and extends upon the findings of Hartl et al. who showed enhanced cytotoxicity when glioma and breast cancer cells were treated with the photosensitizer TPPS2a and Y-90 in its salt form (Y-90Cl_3_)^38^. The time dependent increase in hydroxyl radical generation when nano-photosensitizers were combined with Y-90 microspheres up to 72 h is in keeping with its relatively long half-life of 64.1 h, and it is conceivable that even greater and effective tumoricidal ROS would be realized in the clinical scenario of liver tumor radioembolization where the effective half-life of Y-90 is far greater than its physical half-life.

Contrary to observations in cell-free media, the presence of Y-90 alone without nano-photosensitizer resulted in generation of hydroxyl and mitochondrial superoxide radicals and HCC cell death in vitro and was linearly dependent on Y-90 activity. This is not unexpected given the high energy beta particle interaction in a cellular environment likely results in persistent and elevated downstream oxidative byproducts, mirroring the tumoricidal activity seen with Y-90 alone in the clinic^39^. Interestingly, our results demonstrate enhanced oxidative stress with increasing concentrations of TiO_2_-Tf-TC both on a broad level with increased cellular hydroxyl radical production and specifically at the mitochondrial level with increased mitochondrial superoxide production in the poorly differentiated SNU-387 cells. While both of these oxidative products are to an extent nonspecific given the complex and convergent cellular oxidative pathways, it is in keeping with prior observations that the mitochondria is a therapeutic target of PDT^40^. Importantly, there was significantly decreased viability at Y-90 activities that were non-toxic to both HCC cell lines in vitro alone. This low activity (0.1–0.2 MBq/0.2 mL) corresponds to approximately 25–50 Gy assuming the MIRD model and is well below that prescribed to tumor-bearing liver volumes clinically. With the potential of selective delivery and internalization of nano-photosensitizers to tumor cells in vivo, this may afford tumoricidal action to areas that receive lower Y-90 doses due to microsphere deposition heterogeneity or in patients that are unable to receive higher doses either due to higher risk of background liver toxicities or less favorable vascular conduits^41^. In addition, increased SNU-387 cell death is seen at Y-90 activities up to 0.75 MBq/0.2 mL, which approximately corresponds to 170 Gy according to the MIRD model, also below the perfused liver volume absorbed dose considered for ablative purposes in HCC^7,8,42^. Therefore, implementation of TiO_2_-Tf-TC or other nano-photosensitizers that exhibit tumor retention could conceivably lower this ablative dose threshold for HCC and may show enhanced activity against tumors that require higher doses for response^12^. Importantly, addition of TiO_2_-Tf-TC did not increase oxidative stress or cell death in normal human hepatocytes. Along with the favorable biodistribution profile of TiO_2_-Tf-TC demonstrated in prior work showing minimal off target lung or background liver deposition, we anticipate a low likelihood that this novel treatment strategy would increase the toxicity profile of Y-90^18^.

We did not observe a significant increase in cellular ROS production and cytotoxicity by adding nano-photosensitizers at higher activities of Y-90 (above 0.2 MBq/0.2 mL) in the well differentiated HepG2 cell line, which contrasts with that seen in the poorly differentiated SNU-387 cell line. However, HepG2 cells were more sensitive to the cytotoxic effects of Y-90 alone compared to SNU-387 cells. These two observations may indicate that there may be less room for therapeutic improvements by introducing adjunctive therapies such as nano-photosensitizers in the more sensitive HepG2 cell line. While these results need to be confirmed in a greater number of samples, limited clinical data has shown patients with more poorly differentiated histological grade HCC tumors, as indicated by dual tracer FDG/C-11 acetate positron emission tomography (PET)/CT, had poorer response rates and survival after Y-90 radioembolization than their well-differentiated counterparts^12^. The enhanced oxidative stress and cytotoxicity of poorly differentiated SNU-387 cells with nano-photosensitizers indicates that this novel adjunctive therapy may exert greater therapeutic benefits with certain histologic subtypes, who may already be in critical need for additional therapeutic options. These findings and the substantial genetic heterogeneity of HCC also calls for the careful design of in vivo studies utilizing different orthotopic animal models and patient-derived xenografts of varying malignant potential and genetic profiles to capture which patient cohorts may benefit^43^. This will be critical in properly informing and designing clinical trials.

Regarding implementing depth-independent PDT with Y-90, using its microsphere form (SirSpheres) is advantageous as it is already widely used in the clinic worldwide, avoiding the need to innovate and re-translate the beta-emitting source with often costly and elaborate radiochemistry. Furthermore, we show that activation of nano-photosensitizers is achieved when Y-90 most likely remains extracellular (SirSphere mean diameter 35 μm, with Y-90 permanently imbedded within the resin microsphere preventing leaching), likely as a result of its high decay energy (mean 0.94 MeV, maximum 2.28 MeV) and beta-minus particle tissue penetration of 2–11 mm (mean 2.5 mm)^44^. This obviates the need to reformulate Y-90 to internalize in cancer cells, which is likely required to enable activation of nano-photosensitizers with other lower energy or penetrance radionuclides^18^. Therefore, clinical translation efforts can focus on nano-photosensitizer formulation and can be more readily combined with the clinical workflow of widely implemented Y-90 microspheres either in resin or glass forms. The use of TiO_2_ and TC as nano-photosensitizers is advantageous as both absorb light in the UV range where Cerenkov emission is highest and most efficient, and the former is less susceptible to photobleaching seen with organic photosensitizers^45–48^. In addition, TiO_2_ is a photosensitizer capable of regenerative production of hydroxyl and singlet oxygen radicals^18,49^. This effect can be initiated by irradiation of TiO_2_ with UV-spectrum radiation within 250–350 nm produced by the Cerenkov emission from Y-90 decay. The relative abundance of singlet oxygen and hydroxyl radicals is dependent on the size of the TiO_2_ nanoparticle and the availability of O_2_ with hydroxyl being favored in a hypoxic environment^50^. However, our demonstration of enhanced ROS production with a variety of photosensitizers, including the naturally occurring and clinically used hypericin (Figs. 2A and 3A) provides justification to explore additional nano-photosensitizer formulations. In keeping with prior work, TiO_2_-Tf-TC internalizes into HCC cells in vitro, where PDT is likely to be most efficacious, and may ultimately spatially enhance the tumoricidal activity of Y-90 microspheres^18,51,52^. However, studies examining uptake and retention of nano-photosensitizers over a sufficient time period of Y-90’s decay are needed in clinically relevant in vivo orthotopic models of HCC.

The use and handling of Y-90 microspheres (SirSpheres) for in vitro experiments presents substantial logistical challenges due to their high energy hazards, heterogeneity and microsphere settling, requiring specialized equipment and radiation safety approvals. These hazards proved prohibitive for certain assays that involved luminescence at higher Y-90 activities, flow cytometry, and was a challenge for microscopy studies as the Y-90 had to fully decay (greater than 30 days) before analysis. Settling and heterogeneity of microspheres was controlled for by determining the background luminescence of each experimental condition for cell studies and normalizing to a standard luminescence to activity curve generated with Y-90-Cl_3_. However, the invariably present spatial heterogeneity of Y-90 microspheres in our experimental conditions reflects common working assumptions of microsphere microdosimetry in current practice^14^.

The in vitro data in this study provides motivation to investigate Y-90 microsphere activation of nano-photosensitizers in clinically relevant in vivo HCC models. Given the different effects in two HCC cell lines of different malignant potential and cancer etiology, in vivo models with more aggressive tumor subtypes and patient derived xenografts of similar nature that may be more resistant to Y-90 therapy should be prioritized. Given that administering Y-90 microspheres via the hepatic artery in small animals is resource-intensive and highly technically challenging, efforts to select the correct and most informative in vivo model is of critical importance. These efforts are ongoing. While we demonstrated enhanced cellular oxidative stress and death in HCC cells in vitro compared to Y-90 microspheres alone, this difference was more modest compared to that seen in our cell-free media results and compared to positron beta-emitting radionuclides that do not exhibit cytotoxicity at relevant activities alone in other studies^18,20,22,23^. However, we anticipate the therapeutic effects of Y-90 microspheres combined with nano-photosensitizers to be further apparent in vivo and in the clinic due to the multi-dimensional tumoricidal mechanisms of PDT such as microvascular shutdown and generation of immunogenic cell death which are not captured in in vitro experiments^19^. In addition, we did not assess differences in cell death mechanisms or signaling pathways between Y-90 microspheres alone or combined with nano-photosensitizers, which would likely be more revealing in in vivo HCC models.

In conclusion, stimulation of nano-photosensitizers by Y-90 microspheres generated marked enhancement of ROS production in cell-free media which in turn resulted in enhanced oxidative stress and cell death in highly malignant SNU-387 HCC cells compared to Y-90 microspheres alone. We anticipate these therapeutic effects to be further apparent in vivo and in the clinic due to multi-dimensional tumoricidal mechanisms of PDT, with the ultimate goal of enhancing Y-90 radioembolization for HCC and other liver tumors. In addition, tumor penetration and intracellular uptake of TiO_2_-Tf-TC nano-photosensitizers may improve the spatiotemporal tumoricidal activity of Y-90 microspheres. Applications in clinically relevant in vivo HCC models are underway.

## Supplementary Information

Supplementary Figures.

## Data Availability

The datasets generated during and/or analyzed during the current study are available from the corresponding author on reasonable request.

## References

[1] Bertot LC, Adams LA (2019). Trends in hepatocellular carcinoma due to non-alcoholic fatty liver disease. Expert Rev. Gastroenterol. Hepatol..

[2] Zoller H, Tilg H (2016). Nonalcoholic fatty liver disease and hepatocellular carcinoma. Metabolism.

[3] Bray F (2018). Global cancer statistics 2018: GLOBOCAN estimates of incidence and mortality worldwide for 36 cancers in 185 countries. CA Cancer J. Clin..

[4] Yang JD (2019). A global view of hepatocellular carcinoma: Trends, risk, prevention and management. Nat. Rev. Gastroenterol. Hepatol..

[5] Pfister D (2021). NASH limits anti-tumour surveillance in immunotherapy-treated HCC. Nature.

[6] Ehrhardt GJ, Day DE (1987). Therapeutic use of 90Y microspheres. Int. J. Radiat. Appl. Instrum. B.

[7] Gabr A (2021). Correlation of Y90-absorbed radiation dose to pathological necrosis in hepatocellular carcinoma: Confirmatory multicenter analysis in 45 explants. Eur. J. Nucl. Med. Mol. Imaging.

[8] Salem R (2021). Yttrium-90 radioembolization for the treatment of solitary, unresectable HCC: The LEGACY study. Hepatology.

[9] Garin E (2021). Personalised versus standard dosimetry approach of selective internal radiation therapy in patients with locally advanced hepatocellular carcinoma (DOSISPHERE-01): A randomised, multicentre, open-label phase 2 trial. Lancet Gastroenterol. Hepatol..

[10] Salem R (2010). Radioembolization for hepatocellular carcinoma using Yttrium-90 microspheres: A comprehensive report of long-term outcomes. Gastroenterology.

[11] Spreafico C (2018). Development of a prognostic score to predict response to Yttrium-90 radioembolization for hepatocellular carcinoma with portal vein invasion. J. Hepatol..

[12] Ho CL (2018). Radioembolization with (90)Y glass microspheres for hepatocellular carcinoma: Significance of pretreatment (11)C-acetate and (18)F-FDG PET/CT and posttreatment (90)Y PET/CT in individualized dose prescription. Eur. J. Nucl. Med. Mol. Imaging.

[13] Hermann AL (2020). Relationship of tumor radiation-absorbed dose to survival and response in hepatocellular carcinoma treated with transarterial radioembolization with (90)Y in the SARAH study. Radiology.

[14] Pasciak AS, Bourgeois AC, Bradley YC (2016). A microdosimetric analysis of absorbed dose to tumor as a function of number of microspheres per unit volume in 90Y radioembolization. J. Nucl. Med..

[15] Robertson R (2009). Optical imaging of Cerenkov light generation from positron-emitting radiotracers. Phys. Med. Biol..

[16] Shaffer TM, Pratt EC, Grimm J (2017). Utilizing the power of Cerenkov light with nanotechnology. Nat. Nanotechnol..

[17] Pratt EC, Shaffer TM, Zhang Q, Drain CM, Grimm J (2018). Nanoparticles as multimodal photon transducers of ionizing radiation. Nat. Nanotechnol..

[18] Kotagiri N, Sudlow GP, Akers WJ, Achilefu S (2015). Breaking the depth dependency of phototherapy with Cerenkov radiation and low-radiance-responsive nanophotosensitizers. Nat. Nanotechnol..

[19] Dolmans DE, Fukumura D, Jain RK (2003). Photodynamic therapy for cancer. Nat. Rev. Cancer.

[20] Kotagiri N (2018). Radionuclides transform chemotherapeutics into phototherapeutics for precise treatment of disseminated cancer. Nat. Commun..

[21] Tang R (2020). Osteotropic radiolabeled nanophotosensitizer for imaging and treating multiple myeloma. ACS Nano.

[22] Duan D (2018). Activating TiO_2_ nanoparticles: Gallium-68 serves as a high-yield photon emitter for Cerenkov-induced photodynamic therapy. ACS Appl. Mater. Interfaces.

[23] Kamkaew A (2016). Cerenkov radiation induced photodynamic therapy using chlorin e6-loaded hollow mesoporous silica nanoparticles. ACS Appl. Mater. Interfaces.

[24] Beattie BJ (2012). Quantitative modeling of Cerenkov light production efficiency from medical radionuclides. PLoS One.

[25] Luksiene Z, Kalvelyte A, Supino R (1999). On the combination of photodynamic therapy with ionizing radiation. J. Photochem. Photobiol. B.

[26] Wang GD (2016). X-ray induced photodynamic therapy: A combination of radiotherapy and photodynamic therapy. Theranostics.

[27] Mukerji R (2016). Spatiotemporally photoradiation-controlled intratumoral depot for combination of brachytherapy and photodynamic therapy for solid tumor. Biomaterials.

[28] Zhang C (2015). Marriage of scintillator and semiconductor for synchronous radiotherapy and deep photodynamic therapy with diminished oxygen dependence. Angew. Chem. Int. Ed. Engl..

[29] Caruso S (2019). Analysis of liver cancer cell lines identifies agents with likely efficacy against hepatocellular carcinoma and markers of response. Gastroenterology.

[30] Gill RK, Mitchell GS, Cherry SR (2015). Computed Cerenkov luminescence yields for radionuclides used in biology and medicine. Phys. Med. Biol..

[31] Bancirova M (2011). Sodium azide as a specific quencher of singlet oxygen during chemiluminescent detection by luminol and Cypridina luciferin analogues. Luminescence.

[32] Wang Q (2021). Radioiodinated persistent luminescence nanoplatform for radiation-induced photodynamic therapy and radiotherapy. Adv. Healthc. Mater..

[33] Adachi M (2019). Transferrin receptor 1 overexpression is associated with tumour de-differentiation and acts as a potential prognostic indicator of hepatocellular carcinoma. Histopathology.

[34] Golla K, Cherukuvada B, Ahmed F, Kondapi AK (2012). Efficacy, safety and anticancer activity of protein nanoparticle-based delivery of doxorubicin through intravenous administration in rats. PLoS One.

[35] Mukhopadhyay P, Rajesh M, Yoshihiro K, Hasko G, Pacher P (2007). Simple quantitative detection of mitochondrial superoxide production in live cells. Biochem. Biophys. Res. Commun..

[36] Hirschberg, H.* et al.* Ultra low fluence rate photodynamic therapy: Simulation of light emitted by the Cerenkov effect. in *Optical Techniques in Neurosurgery, Neurophotonics, and Optogenetics* (2014).

[37] Glaser AK, Zhang R, Andreozzi JM, Gladstone DJ, Pogue BW (2015). Cherenkov radiation fluence estimates in tissue for molecular imaging and therapy applications. Phys. Med. Biol..

[38] Hartl BA, Hirschberg H, Marcu L, Cherry SR (2016). Activating photodynamic therapy in vitro with Cerenkov radiation generated from yttrium-90. J. Environ. Pathol. Toxicol. Oncol..

[39] Sgouros G, Bodei L, McDevitt MR, Nedrow JR (2020). Radiopharmaceutical therapy in cancer: Clinical advances and challenges. Nat. Rev. Drug Discov..

[40] Dougherty TJ (1998). Photodynamic therapy. J. Natl. Cancer Inst..

[41] Fox RA (1991). Dose distribution following selective internal radiation therapy. Int. J. Radiat. Oncol. Biol. Phys..

[42] Mazzaferro V (2013). Yttrium-90 radioembolization for intermediate-advanced hepatocellular carcinoma: A phase 2 study. Hepatology.

[43] Ally A (2017). Comprehensive and integrative genomic characterization of hepatocellular carcinoma. Cell.

[44] Westcott MA, Coldwell DM, Liu DM, Zikria JF (2016). The development, commercialization, and clinical context of yttrium-90 radiolabeled resin and glass microspheres. Adv. Radiat. Oncol..

[45] Kumar SG, Devi LG (2011). Review on modified TiO2 photocatalysis under UV/visible light: Selected results and related mechanisms on interfacial charge carrier transfer dynamics. J. Phys. Chem. A.

[46] Davidenko N, Garcia O, Sastre R (2003). The efficiency of titanocene as photoinitiator in the polymerization of dental formulations. J. Biomater. Sci. Polym. Ed..

[47] Potter WR, Mang TS, Dougherty TJ (1987). The theory of photodynamic therapy dosimetry: Consequences of photo-destruction of sensitizer. Photochem. Photobiol..

[48] Gilson RC, Black KCL, Lane DD, Achilefu S (2017). Hybrid TiO_2_-ruthenium nano-photosensitizer synergistically produces reactive oxygen species in both hypoxic and normoxic conditions. Angew. Chem. Int. Ed. Engl..

[49] Linsebigler AL, Lu G, Yates JT (1995). Photocatalysis on TiO_2_ surfaces: Principles, mechanisms, and selected results. Chem. Rev..

[50] Lane DD (2020). Effects of core titanium crystal dimension and crystal phase on ROS generation and tumour accumulation of transferrin coated titanium dioxide nanoaggregates. RSC Adv.

[51] Srivatsan A (2015). Effect of chirality on cellular uptake, imaging and photodynamic therapy of photosensitizers derived from chlorophyll-a. Bioorg. Med. Chem..

[52] Gilson RC, Tang R, Gautam KS, Grabowska D, Achilefu S (2019). Trafficking of a single photosensitizing molecule to different intracellular organelles demonstrates effective hydroxyl radical-mediated photodynamic therapy in the endoplasmic reticulum. Bioconjug. Chem..

