# Supplementary material for: Activation of nano-photosensitizers by Y-90 microspheres to enhance oxidative stress and cell death in hepatocellular carcinoma
Source: Sci Rep. 2022 Jul 26;12:12748. doi: 10.1038/s41598-022-17185-0 (PMC9325688; doi:10.1038/s41598-022-17185-0)
Supplement: Supplementary file 1 — Supplementary Figures. [file 41598_2022_17185_MOESM1_ESM.docx]

**Supplemental Data**

**
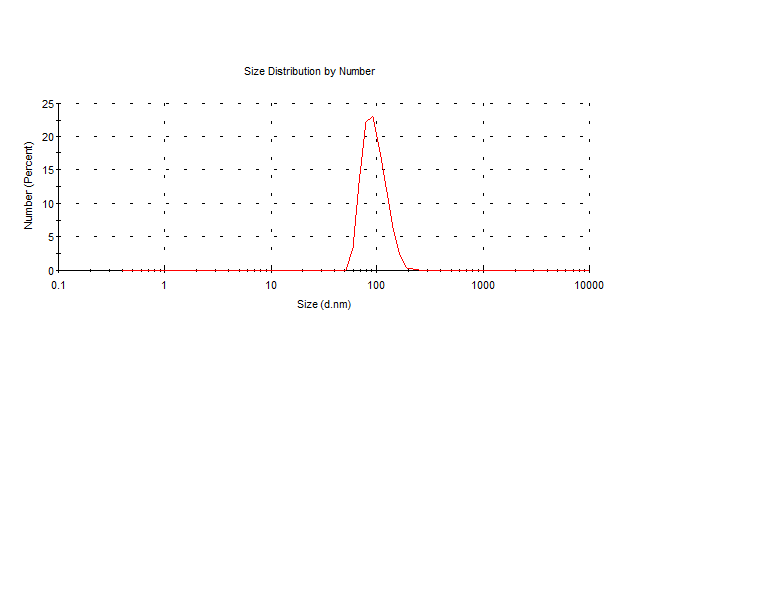
**

**Supplemental Figure 1.** Representative dynamic light scattering (DLS) using a Malvern Zetasizer Nano ZS system of TiO_2_-Tf-TC nano-photosensitizer demonstrating a uniform hydrodynamic diameter of about 100 nm.


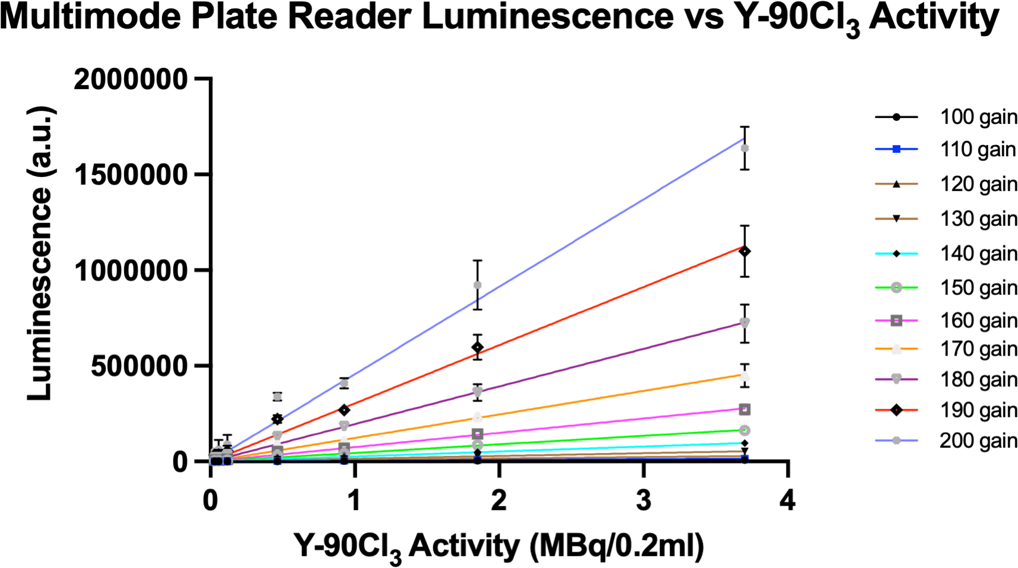


**Supplemental Figure 2**. Luminescence output of increasing Y-90-Cl_3_ activities were obtained from a multimode plate reader. Simple linear regression demonstrated a linear relationship between luminescence output (a.u.) and activity at multiple plate reader gains that was used to determine Y-90 microsphere activities in other experiments. Y90-Cl_3_ was used for this calibration as it could be more predictably handled and diluted compared to the heterogenous Y-90 microspheres. Values represent mean +/- standard deviation of three measurements.


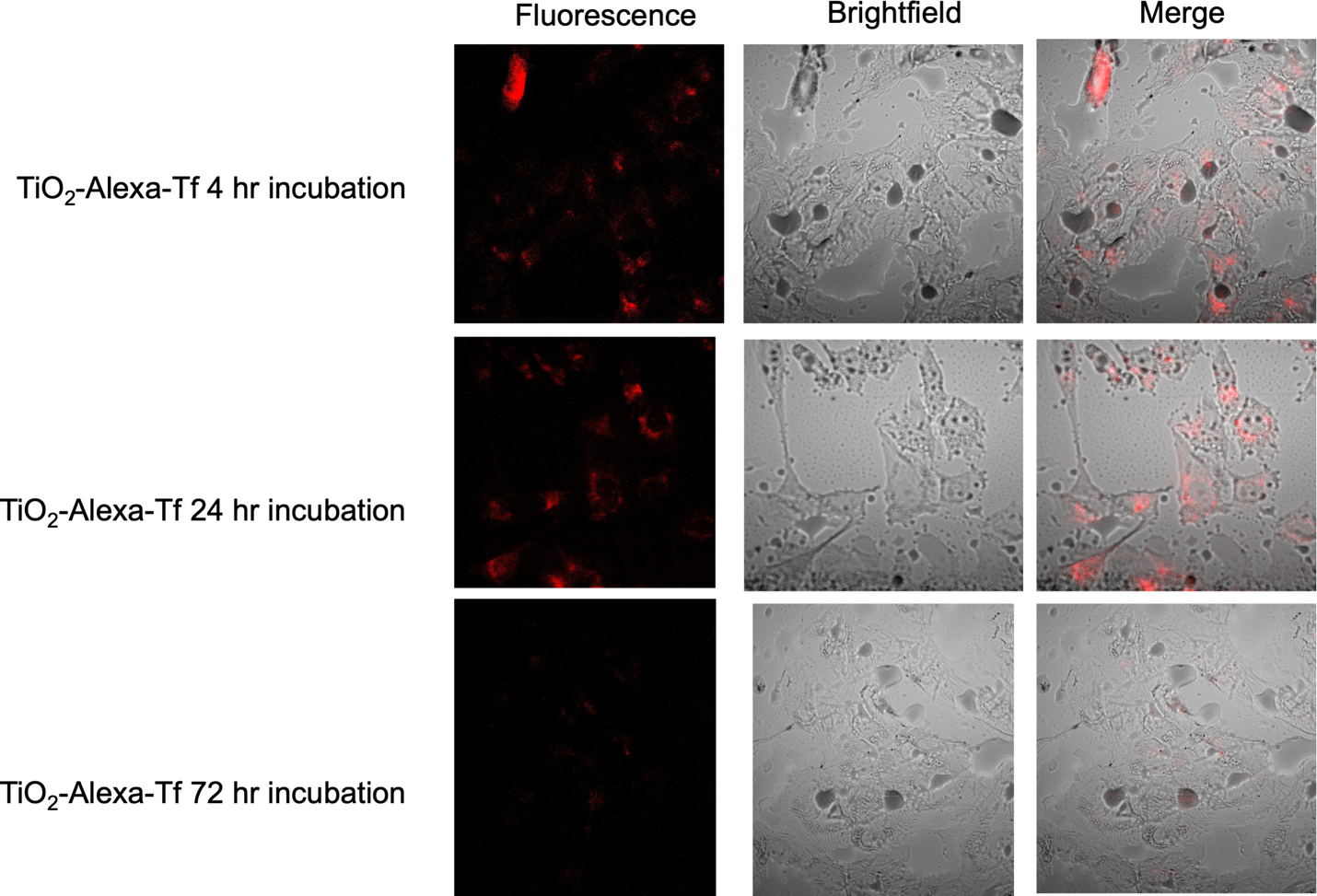


**Supplemental Figure 3. Cellular uptake of a targeted nano-photosensitizer TiO_2_-Tf.** SNU-387 HCC cells incubated with transferrin labelled TiO_2_-Tf conjugated to the fluorescent probe AlexaFluor-680 (TiO_2_-AlexaTf) demonstrates intracellular uptake that is retained over time. High intracellular uptake of nano-photosensitizer is seen at 24 hours. Nano-photosensitizers are retained at 72 hours although to a lesser extent compared to earlier timepoints.


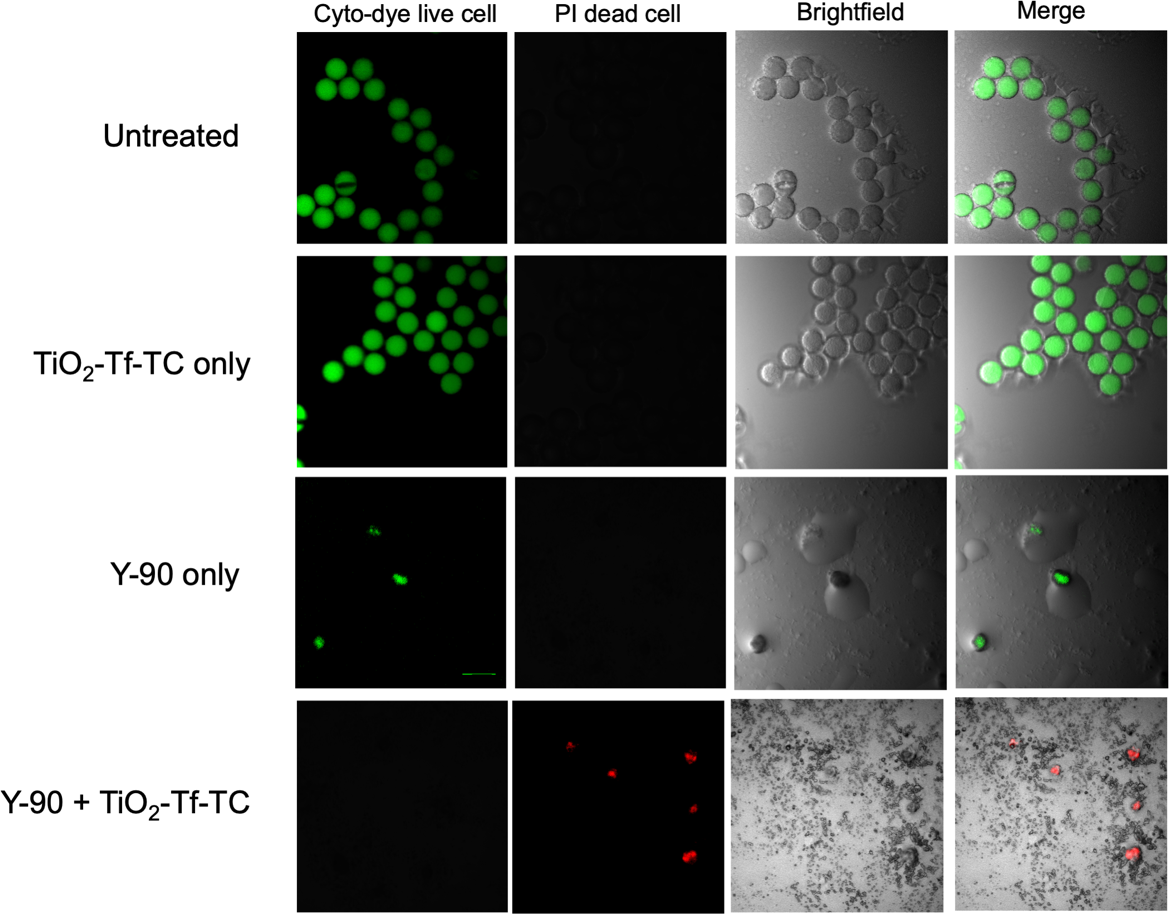


**Supplemental Figure 4.** Representative live/dead confocal microscopy of SNU-387 cells under various treatment conditions. Cells that were either untreated (control) or treated with TiO_2_-Tf-TC only show predominantly live cells without any death. Cells treated with Y-90 microspheres alone (0.52 MBq/0.2ml) show only a few lives cells on a background of cellular debris likely from prior cell death. Cells treated with combined Y-90 microspheres and TiO_2_-Tf-TC demonstrated no live cells, with an abundance of cellular debris and necrotic cells (stained by propidium iodide), indicating enhanced cytotoxicity with this combination.
